# Cav 1.3 damages the osteogenic differentiation in osteoporotic rats by negatively regulating Spred 2‐mediated autophagy‐induced cell senescence

**DOI:** 10.1111/jcmm.15978

**Published:** 2020-10-30

**Authors:** Ping Fan, Dan Pu, Xiaohong Lv, Nan Hu, Xiuyuan Feng, Zhiming Hao, Yining Sun, Lan He

**Affiliations:** ^1^ Department of Rheumatism and Immunology the First Affiliated Hospital of Xi’an Jiaotong University School of Medicine Xi’an China

**Keywords:** BMSCs, Cav 1.3, osteogenic differentiation, Spred 2

## Abstract

Cav 1.3 can affect the classical osteoclast differentiation pathway through calcium signalling pathway. Here, we performed cell transfection, real‐time fluorescence quantitative PCR (qPCR), flow cytometry, SA‐β‐Gal staining, Alizarin Red S staining, ALP activity test, immunofluorescence, Western blot and cell viability assay to analyse cell viability, cell cycle, osteogenesis differentiation and autophagy activities in vitro. Meanwhile, GST‐pull down and CHIP experiments were conducted to explore the influence of Cav 1.3 and Sprouty‐related EVH1 domain 2 (Spred 2) on bone marrow–derived mesenchymal stem cells (BMSCs). The results showed that OS lead to the decreased of bone mineral density and differentiation ability of BMSCs in rats. Cav 1.3 was up‐regulated in OS rats. Overexpression of Cav 1.3 inhibited the activity of BMSCs, the expression of alkaline phosphatase (ALP), runt‐related transcription factor 2 (RUNX2) and osteocalcin (OCN), as well as promoted the cell cycle arrest and senescence. Furthermore, the negative correlation between Cav 1.3 and Spred 2 was found through GST‐pull down and CHIP. Overexpression of Spred 2 increased the expressions of microtubule‐associated protein 1 light chain 3 (LC3) and Beclin 1 of BMSCs, which ultimately promoted the cell activity of BMSCs and ALP, RUNX2, OCN expression. In conclusion, Cav 1.3 negatively regulates Spred 2‐mediated autophagy and cell senescence, and damages the activity and osteogenic differentiation of BMSCs in OS rats.

## 1 BACKGROUND

OS is a systemic bone disease that potentially threatens the health of the elderly and post‐menopausal women. Due to the aggravation of the ‘degree of aging’, the prevalence of osteoporosis is as high as 34.65% in people over 50 years old in China, and the prevalence of women is higher than that of men.^1^ Reduced bone mass and degenerated bone microstructure not only cause spontaneous fractures, but also lead to other diseases.^2^ Currently, osteoporosis is mainly based on medical treatment of symptoms and complications. However, it urgently needs to find a more effective treatment due to the high cost, poor treatment effect and adverse reactions. Gene therapy, as a treatment method emerging in recent years, can be targeted to gene defects and diseased tissues.^3^ It is known that the differentiation ability of osteoclasts and osteoblasts is an important factor affecting the process of osteoporosis.^4^, ^5^ The amount of bone formed in old age is less than the amount of bone absorbed, which can lead to osteoporosis. However, its exact pathological mechanism remains unclear.

BMSCs are a kind of pluripotent cells derived from bone marrow tissues. They have self‐renewal and multi‐directional differentiation capabilities, and can be differentiated into various mesoderm cell types such as osteoblasts, chondrocytes and fat cells.^6^, ^7^ In the dynamics of bone, the osteogenesis process is initiated by the proliferation/mineralization of BMSCs after they are recruited to the site of bone remodelling.^8^ Therefore, the osteogenic differentiation ability of BMSCs is crucial in bone remodelling. However, aging will not only reduce the number of BMSCs, but also reduce its osteogenic differentiation ability through overactivated autophagy.^9^ Especially in senile osteoporosis, cell senescence may be another factor affecting osteoblast differentiation.

Autophagy is a conservative steady‐state mechanism of lysosomal degradation of foreign substances in cells. When autophagy occurs, autophagosomes that form double‐membrane or multi‐membrane vesicles play a role in the cell. Studies have shown that Spred protein, as a negative regulator of growth factors, can induce caspase‐dependent and autophagy‐dependent cell death.^10^ And autophagy‐related proteins Atg5, Atg7, Atg4B and LC3 play an active role in osteoclast differentiation and bone resorption,^11^, ^12^ and ultimately affect the dynamic balance of bone. But its effect on osteoblast differentiation is not clear.

Previous studies have shown that Cav 1.3 can promote osteoclast differentiation from osteoclast precursor cells to osteoclasts and bone resorption. Cav 1.3 may affect the classical osteoclast differentiation pathway through calcium signalling pathway.^13^ However, its differentiation effect on osteoblasts is not clear. In this study, we focused on investigating the interaction between Cav 1.3 and Spred 2, and their effects on autophagy, cell senescence and osteogenic differentiation of BMSCs derived from OS rats.

## 2 MATERIAL AND METHODS

### 2.1 Establishment of OS rat model

Thirty Sprague‐Dawley (SD) rats of 3 months old, 6 rats in each group, were fed with 80 mg/(kg/d) for 15 days by retinoic acid (dissolved in vegetable oil) for 15 days. The bone density of the living femur was measured. Meanwhile, comparing the bodyweight of rats before and after gavage, the bone density was significantly reduced and the weight loss indicated that the model was established successfully. Normal rats served as a control group.

### 2.2 BMSCs cell isolation, culture and osteogenic induction

3‐week‐old SD rats were selected in the OS group and the control group. The rats were injected with excessive chloral hydrate and cut the femurs of the hind limbs, and washed the limbs with phosphate‐buffered saline (PBS) three times. The marrow cavity was flushed repeatedly by the culture medium to rinse out the cells. Subsequent the cells were centrifugated at 900 *g* for 5 minutes and inoculated into a Petri dish at 37°C, 5% CO^2^. The medium was changed every 3 days and passaged once every 7 days.

Induction of osteogenic differentiation of BMSCs cultured to 3 generations: 5 × 10^3^ cells/cm^2^ cells were seeded in a six‐well plate, and 10% foetal bovine serum (FBS)‐added α‐minimum eagle's medium (MEM) culture medium containing 50 μmol/L ascorbic acid, 10 mm β‐glycerol phosphate and 100 nm Dexamethasone. The medium was changed every 3 days until 28 days.

### 2.3 Groups

Control‐BMSCs group: normal rats‐derived BMSCs. OS‐BMSCs group: OS rats‐derived BMSCs. OE‐Cav 1.3: overexpression of Cav 1.3 in OS‐BMSCs group. OE‐NC: overexpression of Cav 1.3 or Spred 2 in control‐BMSCs group. OE‐Spred 2: overexpression of Spred 2 in OS‐BMSCs group. OE‐Cav 1.3 + Spred 2: overexpression of Cav 1.3 and Spred 2 in OS‐BMSCs group. sh‐Cav 1.3: inhibition of Cav 1.3 in OS‐BMSCs group. sh‐Spred 2: inhibition of Spred 2 in OS‐BMSCs group. sh‐NC: inhibition of Cav 1.3 or Spred 2 in OS‐BMSCs group. OE‐Spred 2 + 3‐MA: overexpression of Spred 2 in OS‐BMSCs group and add 5 mmol/L/μL of 3‐Methyladenine (3‐MA).

### 2.4 Cell counting kit 8 (CCK8) test to detect the proliferation of cells

The 10^5^/ml cell suspension (100 μL/well) was inoculated in a 96‐well plate, and the culture plate was placed in the incubator for pre‐culture (37°C, 5% CO^2^). 10 μL of CCK8 solution was added into the each well, and the plate incubated at 37 degrees for 1‐4 hours. Finally, the absorbance at 450 nm was measured with a microplate reader. The autophagy pathway was inhibited by 5 mmol/L/μL of 3‐MA and detect cell activity were detected by a microplate reader.

### 2.5 SA‐β‐Gal staining to detect cell senescence

The cells in the normal and OS groups in the 6‐well plate were washed three times with PBS. One millilitre of staining fixative was added to the 6‐well plate. The cells were fixed at room temperature for 20 minutes. After washing with PBS, the staining solution was added and incubated at 37 degrees overnight. The stained cells were observed with an optical microscope, and the positive rate was counted. Ten fields were selected under the microscope. The total number and positive number were counted (stained and developed in the cytoplasm). The number of cells in each field was controlled within 100‐150.

### 2.6 Alizarin Red S staining

The cells of the normal group and OS composed of bone differentiation were fixed for 30 minutes, washed with PBS 3 times and added with alizarin red dye for 5 minutes, washed with PBS, and finally observed under an optical microscope.

### 2.7 ALP activity test

After the osteogenic differentiation culture on the 48‐well plate, the medium was removed and washed 3 times with PBS. In the normal group and the OS group, 0.05% Triton X (lysed cell membrane) was added to each well to perform freeze‐thaw‐freeze‐thaw. After centrifugation at 4 degrees/15 000 rpm, the supernatant was collected. After 100 μL substrate and 20 μL sample were added to 96‐well plate and shaken for 1 minutes, and incubated at 37 degrees for 15 minutes, 80 μL top solution was added to stop the reaction. The microplate reader measures the absorbance at 450 nm.

### 2.8 Immunofluorescence

The cells in the normal group and the OS group were fixed for 30 minutes; they were washed three times with PBS. 5% BSA was added to the fixed cells to block the cells for 30 minutes and then washed with PBS three times. Primary antibody (LC 3, 1:500, ab192890, Abcam) was added to the blocked cells and incubated at 4 degrees in the dark overnight. After the primary antibody is incubated, the cells were washed with PBS 3 times. Secondary antibody (1:1000, ab150113, Abcam) was added and incubated at room temperature for 30 minutes, and finally observed the cells under an optical microscope.

### 2.9 Flow cytometry analysis

The cells in the normal group and OS group were digested with trypsin and centrifuged at 900 *g* for 5 min. The PBS was added to resuspend the cells. 0.5 mL of 50 μg/mL propidium iodide (PI) solution were added to 1*105 cells and stained in the dark for 30 minutes at room temperature. The cells in the centrifuge tube were filtered with a 300 μm nylon mesh into a new centrifuge tube to make a single cell suspension and inserted it into a flow cytometer.

### 2.10 Total RNA extraction, reverse transcription and qPCR

The cells in the normal and OS groups were lysed with Trizol, chloroform was used to extract the total RNA, and the kit was used to reverse the RNA to cDNA. SLAN fluorescence quantitative PCR instrument was used to detect the expression of Cav1.3 and Spred 2 in BMSCs after osteogenic induction. The expression of osteogenic differentiation‐related genes ALP, RUNX2 and OCN and the expression of autophagy‐related genes LC3 II/I and Beclin1 were detected.

Gene sequence: Cav1.3: Forward 5′‐GGAGGCTTTGATGTCGAAGCCCT‐3′, Reverse 5′‐CCTCCGAAACTACAGCTTCGGGAG‐3′. Spred2: Forward 5′‐ GGA GGCTTTGATGTCGAAGCCCT‐3′, Reverse 5′‐ CCTCCG AAACTACAGCTTCGG GAG‐3′. ALP: Forward 5′‐TCACTTCCGCCCGGAACCCT‐3′. Reverse 5′‐TGTCCT GCCGGCCCAAGAGA‐3′. RUNX2: Forward: 5′‐GCGGACGAGGCAAGAGTT‐3′ Reverse: 5′‐TTGGTGCTGAGTTCAGGGAG‐3. OCN: Forward: 5′‐TGAGGACC CTCTCTCTGCTC‐3′, Reverse: 5′GGGCTCCAAGTCCATTGTT‐3′. GAPDH: Forward 5′‐GTTTACATGTTCCAATATG‐3′, Reverse 5′‐ GTGGGTGTCGCTGTTG AAG‐3′.

### 2.11 Cell transfection

The overexpression plasmid and interference plasmid of Cav 1.3 gene and Spred 2 were purchased from Shanghai Bioengineering Co., Ltd. and named as OE‐Cav 1.3, sh‐Cav 1.3, OE‐Spred 2 and sh‐Spred 2. The OE‐Cav 1.3, sh‐Cav 1.3, OE‐Spred 2 and sh‐Spred 2 plasmids were transfected into the normal group and OS group cells using Lipofectamine3000 (Invitrogen, USA), and the transfection and expression were checked by light microscope and PCR after 24 hours.

### 2.12 Western Blot analysis

Cells in each group were collected and 200 μL of cell lysate was added to each well, and the cells were lysed on ice for 1 hour. Subsequently, it was centrifuged at 11260 *g* for 15 minutes at 4°C. The centrifuged supernatant was transferred to a clean centrifuge tube. The protein concentration of each group was determined using the bicinchoninic acid (BCA) protein quantification kit and stored at −80°C. In Western blot electrophoresis, the loading concentration was 30‐100 micrograms per well. After sodium dodecyl sulphate polyacrylamide gel electrophoresis (SDS‐PAGE) electrophoresis, the membrane was transferred and blocked. P16, P21, P53, Cav 1.3, Spred 2, LC 3I, LC3 II and Beclin1, SA‐β‐Gal, GST, GAPDH (1:500, antimouse, Abcam) were diluted and incubated at 4 degrees overnight. The secondary antibody was incubated in the dark at room temperature for 30 minutes (1:1000, Abcam). Developers are used for development and photography.

### 2.13 GST‐pull down

Cav 1.3 recombinant protein containing GST tag and Spred 2 protein containing His tag were purchased from Shanghai Bioengineering Co., Ltd. GST‐Cav1.3 recombinant protein was mixed with His‐Spred 2 protein in vitro, and 20‐30 microlitres of His‐Spred 2 protein was added to the Sepharose 4B suspension combined with GST‐Cav1.3 protein, and incubated for 6 hours on a 4 degree shaker. The precipitate was collected by centrifugation at 2700 *g* for 3 minutes at 4 degrees Celsius and washed 5 times with PBS. 30 microlitres of loading buffer was added for SDS‐PAGE electrophoresis to detect the interaction between Cav 1.3 protein and Spred 2 protein.

### 2.14 CHIP

Cells in normal and OS groups were fixed with 4% paraformaldehyde for 30 minutes, collected and sonicated. CAV 1.3 protein antibody was added, followed by 60 μL of protein A agarose DNA and incubated at 4 degrees for 2 hours. After centrifugation, the supernatant was removed and washed. The complex was de‐crosslinked, and the resulting complex was purified. Finally, the obtained complex was tested by PCR.

### 2.15 Statistical analysis

The data were analysed using SPSS 19.0 software, and all data were expressed as mean ± standard deviation (SD). Prism 6.0 software was used for t test and one‐way ANOVA or Student's *t* test for differences between groups and within groups. *P* < .05 was considered to have a significant statistical difference. And all experiments were carried out independently three times.

## 3 RESULTS

### 3.1 The expression of Cav1.3 in BMSCs derived from OS rats increased while the expression of Spred2 decreased

**FIGURE 1:**
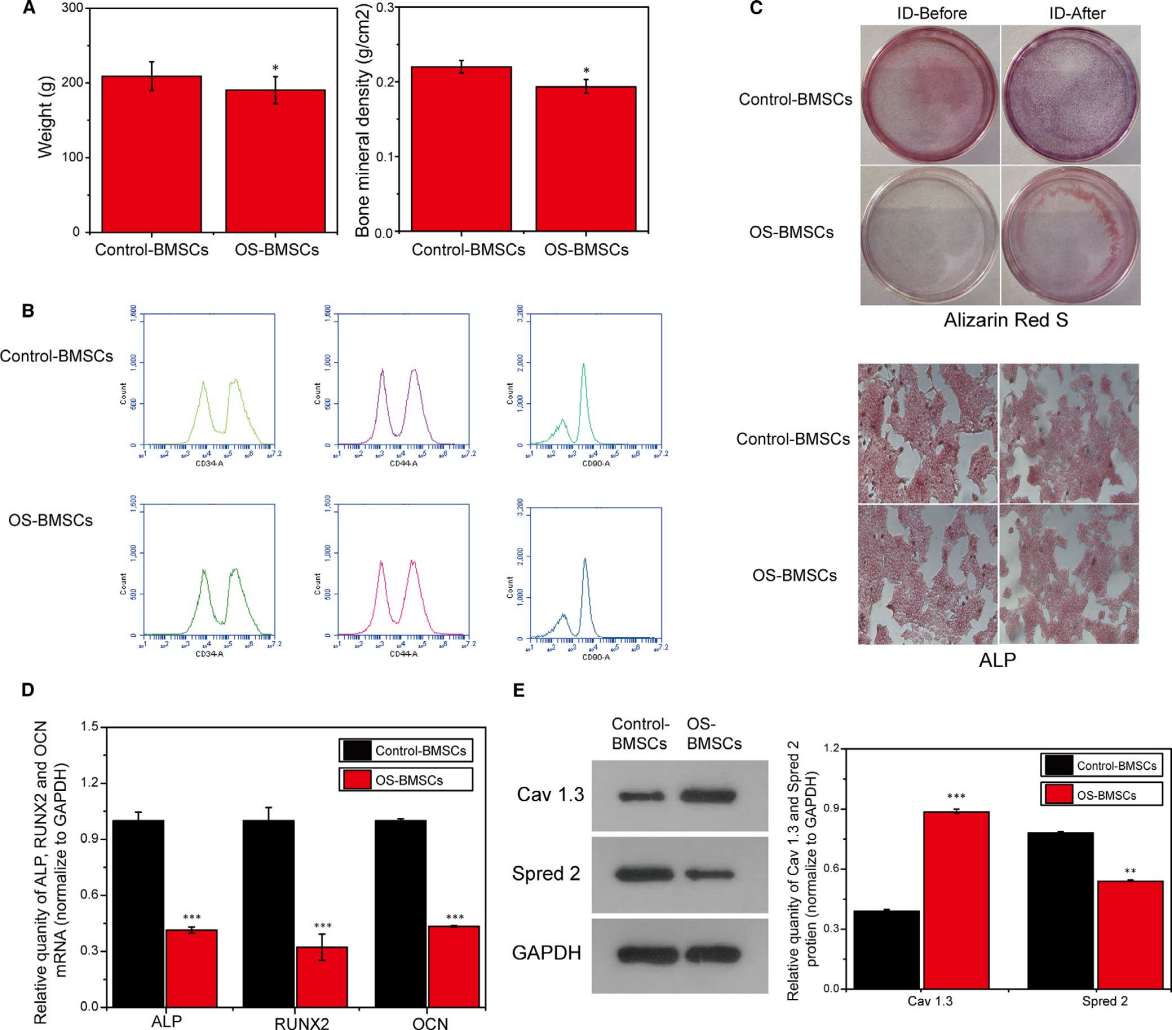
The expression of Cav1.3 in BMSCs derived from OS rats increased while the expression of Spred2 decreased. A, Comparison of bone density and bodyweight of rats in normal group and OS group. B, Flow cytometric identification of BMSCs. C, Alizarin red S staining and ALP identification of osteogenic differentiation of BMSCs. D, Expression of BMSCs osteogenic differentiation‐related genes ALP, RUNX2, OCN. E, The expression of Cav1.3 and Spred2 in the normal group and OS group by Western blotting. *, *P* < .05; **, *P* < .01; ***, *P* < .001

In this study, the bone density and bodyweight of rats in the normal group and the OS group were first compared. As shown in Figure 1A, the bone density and bodyweight of the rats in the OS group were significantly lower than those in the normal group. Subsequently, BMSCs were extracted from the normal group and the OS group, and the surface markers CD34 ^+^, CD44 ^+^ and CD90 ^+^ of BMSCs were identified by flow cytometry in the third generation. The positive expression of CD34 ^+^, CD44 ^+^ and CD90 ^+^ was as high as 90%, indicating that the extracted and cultured BMSCs can be used for follow‐up experiment (Figure 1B). We simultaneously induced osteogenic differentiation (ID) of two groups of BMSCs. Alizarin red S staining and ALP identification showed that the osteogenic differentiation ability of BMSCs in the OS group was significantly lower than that of the normal group (Figure 1C). PCR results showed that the expression of osteogenic differentiation‐related genes ALP, RUNX2 and OCN in OS group was also lower than that in normal group, indicating that the differentiation ability of BMSCs in OS group decreased. Western blotting results showed that the expression of Cav1.3 in BMSCs derived from OS rats increased while the expression of Spred2 decreased, and the difference was statistically significant (*P* < .05).

### 3.2 The overexpression of Cav1.3 inhibited the osteogenic differentiation ability of BMSCs in OS group

**FIGURE 2:**
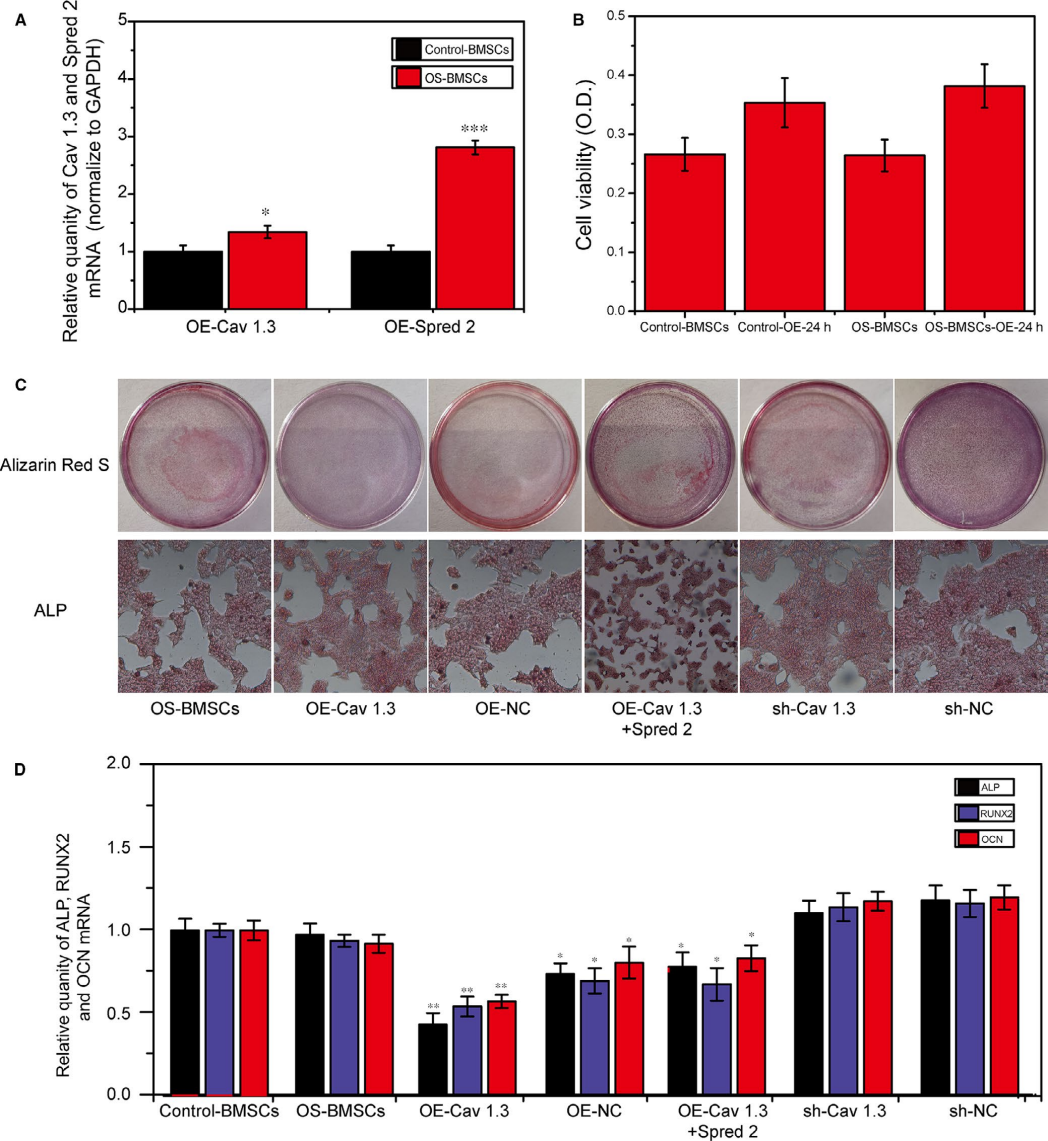
Overexpression of Cav1.3 inhibited the osteogenic differentiation ability of BMSCs in OS group. A, PCR result of overexpression of Cav1.3 and Spred2. B, BMSCs viability detection after overexpression. C, Detection of Alizarin Red S and ALP for osteogenic differentiation of BMSCs overexpressing and inhibiting Cav1.3 and SPRED2. D, PCR results of osteogenic differentiation‐related genes ALP, RUNX2 and OCN. *, *P* < .05; **, *P* < .01; ***, *P* < .001

In order to explore the effects of Cav1.3 and Spred2 on the osteogenic differentiation of BMSCs, the Cav1.3 and Spred2 were overexpressed and the expression of Cav1.3 was inhibited in two groups. Figure 2A showed that the Cav1.3 and Spred2 genes were successfully overexpressed. We tested the cell viability of the two groups of BMSCs before and after transfection, and the results showed that the cell viability was not affected after transfection (Figure 2B). Alizarin Red S and ALP osteogenic differentiation identification showed that the overexpression of Cav1.3 significantly reduced the osteogenic differentiation ability of BMSCs in the OS group (*P* < .05), manifested by low calcium mineralization ability and decreased osteogenic differentiation‐related genes. And the overexpression of Cav1.3 and Spred2 at the same time also impaired the osteogenic differentiation ability of BMSCs in the OS group, but their osteogenic differentiation ability was higher than that of OE‐Cav1.3. On the contrary, the calcium mineralization ability and the expression of osteogenic differentiation‐related genes in the sh‐Cav1.3 group were increased, which indicates that the high expression of Cav1.3 can significantly inhibit the osteogenic differentiation ability of BMSCs (Figure 2C,D).

### 3.3 Cav1.3 inhibits the autophagy ability of BMSCs and Spred2 promotes the autophagy ability of BMSCs

**FIGURE 3:**
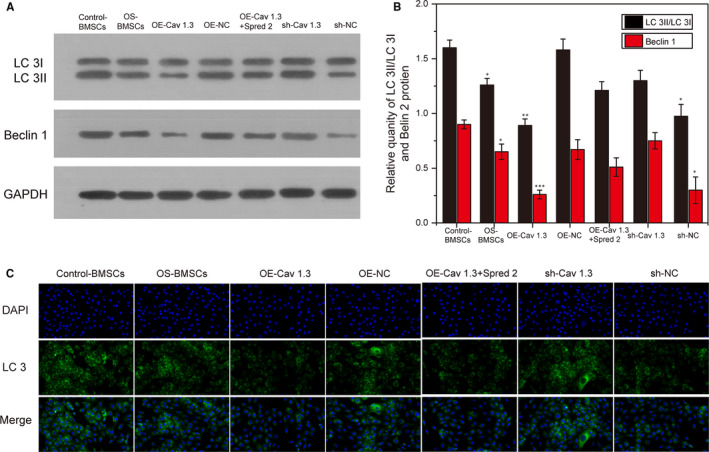
Cav1.3 inhibits the autophagy ability of BMSCs and Spred2 promotes the autophagy ability of BMSCs. A, Beclin 1 and LC 3I/LC 3II protein expression levels after overexpression and suppression of Cav1.3 and Spred2. B, Immunofluorescence detection of overexpression and inhibition of Cav1.3 and Spred2 autophagy levels of BMSCs. Green: LC 3II expression. Blue: DAPI nuclear staining. *, *P* < .05; **, *P* < .01; ***, *P* < .001

In order to explore the effect of Cav1.3 and Spred2 on the autophagy level of BMSCs, we overexpressed and inhibited the expression of Cav1.3 and Spred2 in BMSCs. The results showed that the expression of Beclin 1 and LC 3I/LC 3II in the OE‐Cav1.3 group was significantly reduced. On the contrary, after Cav1.3 was inhibited, the expression levels of Beclin 1 and LC 3I/LC 3II were significantly higher than those in the OE‐Cav1.3 (*P* < .05). And the expression of Beclin 1 and LC 3I/LC 3II in OE‐Cav1.3 + Spred2 group was higher than those of the OE‐Cav1.3 group but lower than the OE‐NC group (Figure 3A,B). Conversely, inhibiting Cav1.3 increased the autophagy level of BMSCs. Similarly, immunofluorescence detection results showed that the expression of Beclin 1 and LC 3I/LC 3II was the lowest in OE‐Cav1.3 and OE‐Cav1.3 + OE‐NC (Figure 3C). These results showed that the highly expressed Cav1.3 can inhibit the autophagy ability of BMSCs and Spred2 promotes the autophagy ability of BMSCs.

### 3.4 Cav1.3 promoted the cell cycle arrest and cell senescence of BMSCs, while Spred2 reduced the cell cycle arrest and cell senescence of BMSCs

**FIGURE 4:**
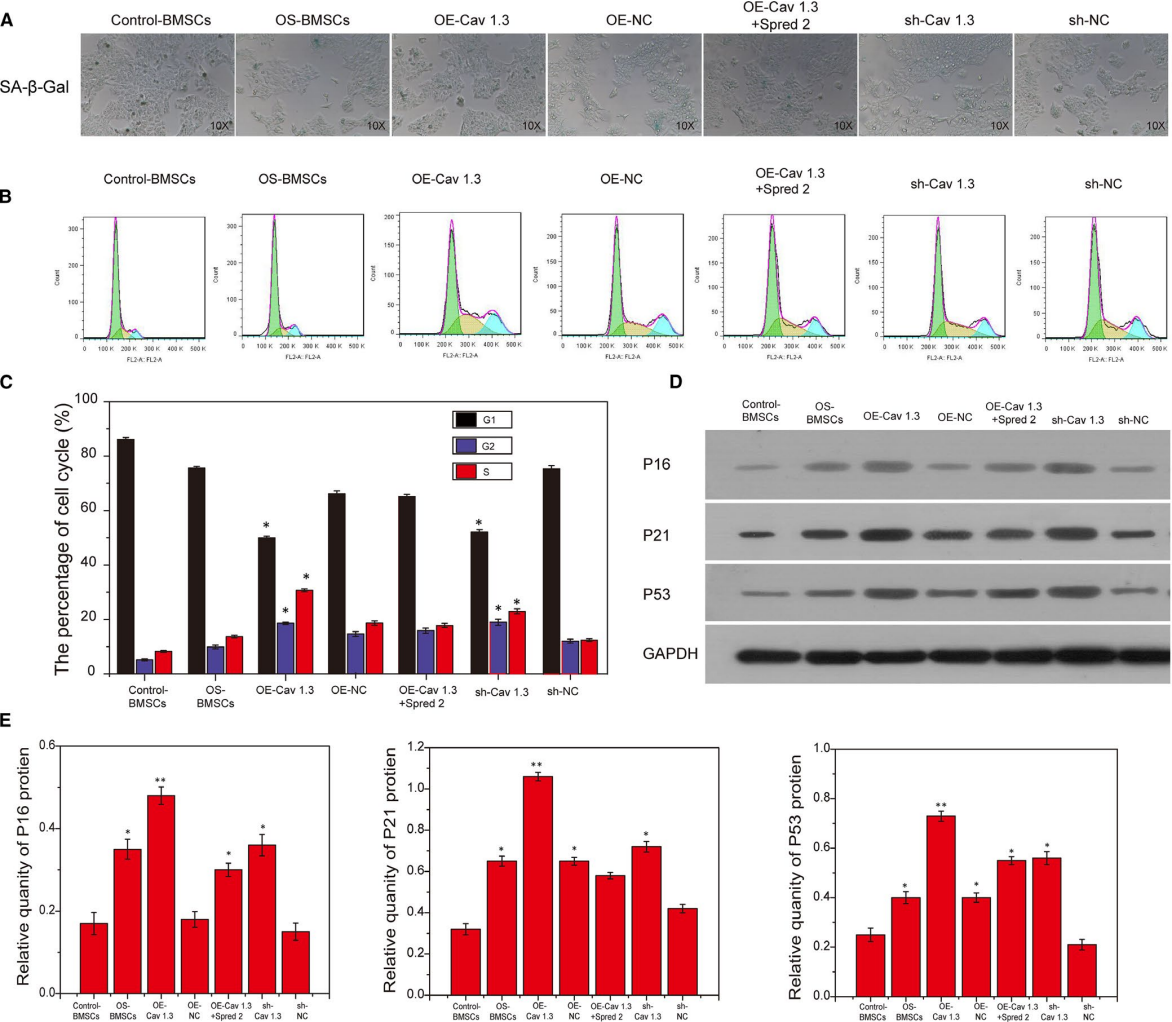
Cav1.3 promoted the cell cycle arrest and cell senescence of BMSCs. A, The senescence degree of different groups of BMSCs detected by SA‐β‐Gal staining. B,, Flow cytometry detected the cell cycle of BMSCs in all groups. C, Statistical analysis of cell cycle. D, The expression of P16, P21 and P53 proteins tested by Western blotting. E, Statistical analysis of Western blotting results. *, *P* < .05; **, *P* < .01; ***, *P* < .001

In order to explore the effects of Cav1.3 and Spred2 on the senescence and cell cycle of BMSCs, we overexpressed and inhibited the expression of Cav1.3 and Spred2 in BMSCs. Cell senescence was detected by SA‐β‐Gal staining and the results showed that the OE‐Cav1.3 group had the highest degree of cell senescence while the sh‐Cac1.3 group had the lowest degree of senescence. The senescence of cells in the OE‐Cav1.3 + Spred2 group was lower than that in the OE‐Cav1.3 group (Figure 4A). Similarly, flow cytometry was used to detect the cycles of different groups of cells. The S phase of BMSCs in the OE‐Cav1.3 group reached 30%, while the S phase of the sh‐Cac1.3 group was the lowest, only 7% (Figure 4B,C). Subsequently, this study detected the expression of cell cycle‐related proteins P16, P21 and P53 in different groups of cells. The expression of P16, P21 and P53 proteins in the OE‐Cav1.3 group was the highest, and the sh‐Cav1.3 group had the lowest (Figure 4D,E). These results indicated that the highly expressed Cav1.3 can promote the senescence and cell cycle arrest of BMSCs in the OS group.

### 3.5 Cav1.3 and Spred2 proteins bind to each other and Cav1.3 negatively regulates spred2

**FIGURE 5:**
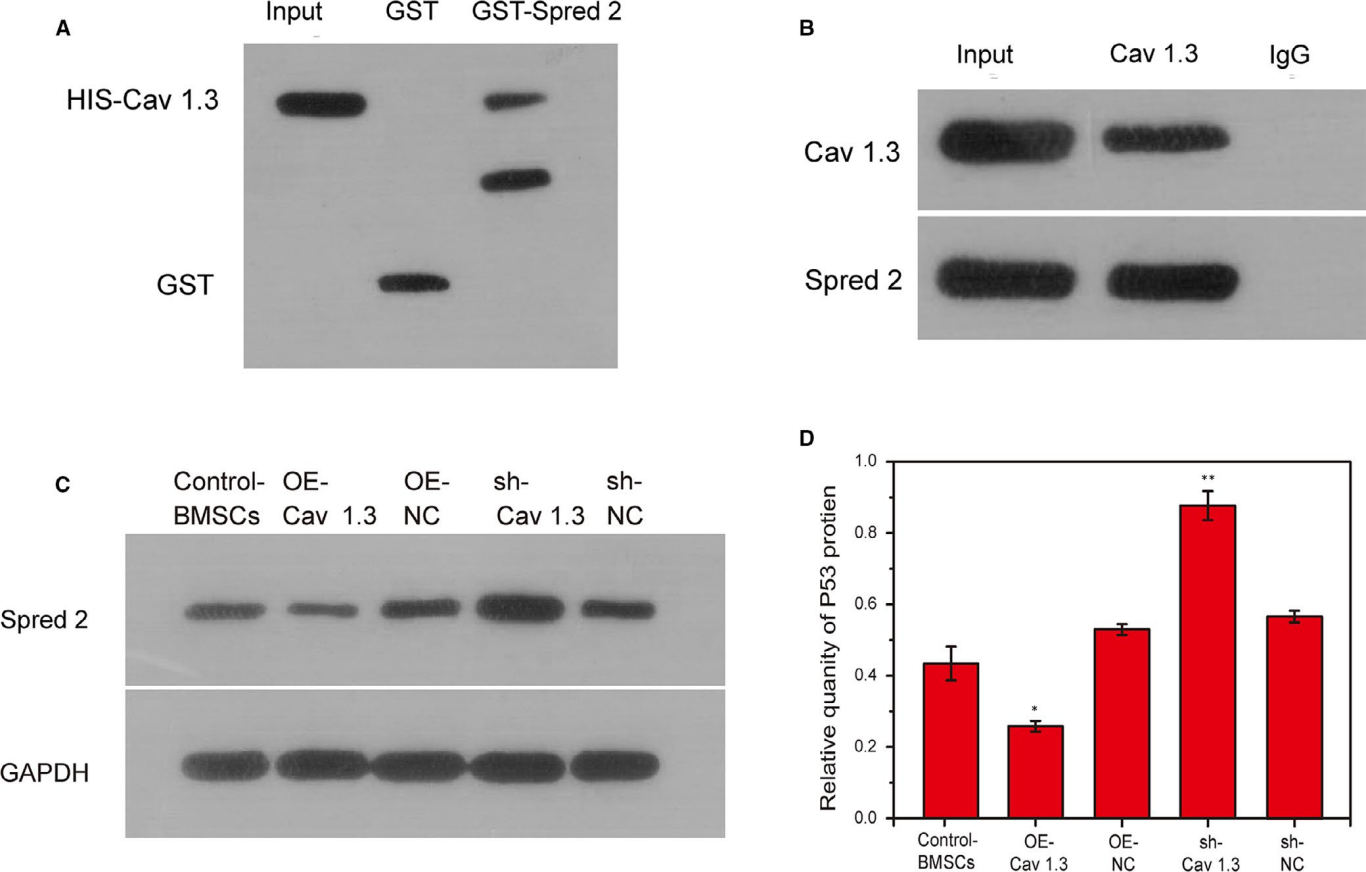
Cav1.3 and Spred2 proteins bind to each other and Cav1.3 negatively regulates spred2. A, GST‐pull down detected the relationship between Cav1.3 and Spred2. B, CHIP tested the relationship between Cav1.3 and Spred2. C, The expression of Spred2 after overexpression of Cav1.3 was detected by Western blotting. D, Statistical analysis of Western blotting results. *, *P* < .05; **, *P* < .01; ***, *P* < .001

In this study, the Cav1.3 gene was overexpressed and inhibited, respectively, and the relationship between Cav1.3 and Spred2 was explored using GST‐pull down and CHIP methods. The results showed that the OE‐Cav 3.1 group had a large amount of Spred2 expression, while the GST control group had no Spred2 expression, indicating that the interaction between Cav1.3 and Spred2 exists. Here, input is a positive control, and GST is a negative control. 40% of the target protein Spred2 was expressed, indicating that Cav1.3 and Spred2 have a relatively strong interaction (Figure 5A). CHIP analysis showed that the input positive control group had rich expression of Cav1.3 and Spred2, while the expression of IgG negative control was less than 0.1%. The degree of enrichment of Cav1.3 and Spred2 target protein was between the input positive control and IgG negative control. The results were credible, and there was a strong relationship between Cav1.3 and Spred2 (Figure 5B). Overexpression of Cav1.3 reduced the expression of Spred2 while the expression of Spred2 increased in the sh‐Cav1.3 group (Figure 5C,D).

### 3.6 Spred2 promoted the activity, autophagy and osteogenic differentiation of BMSCs derived from OS rats by activating autophagy

**FIGURE 6:**
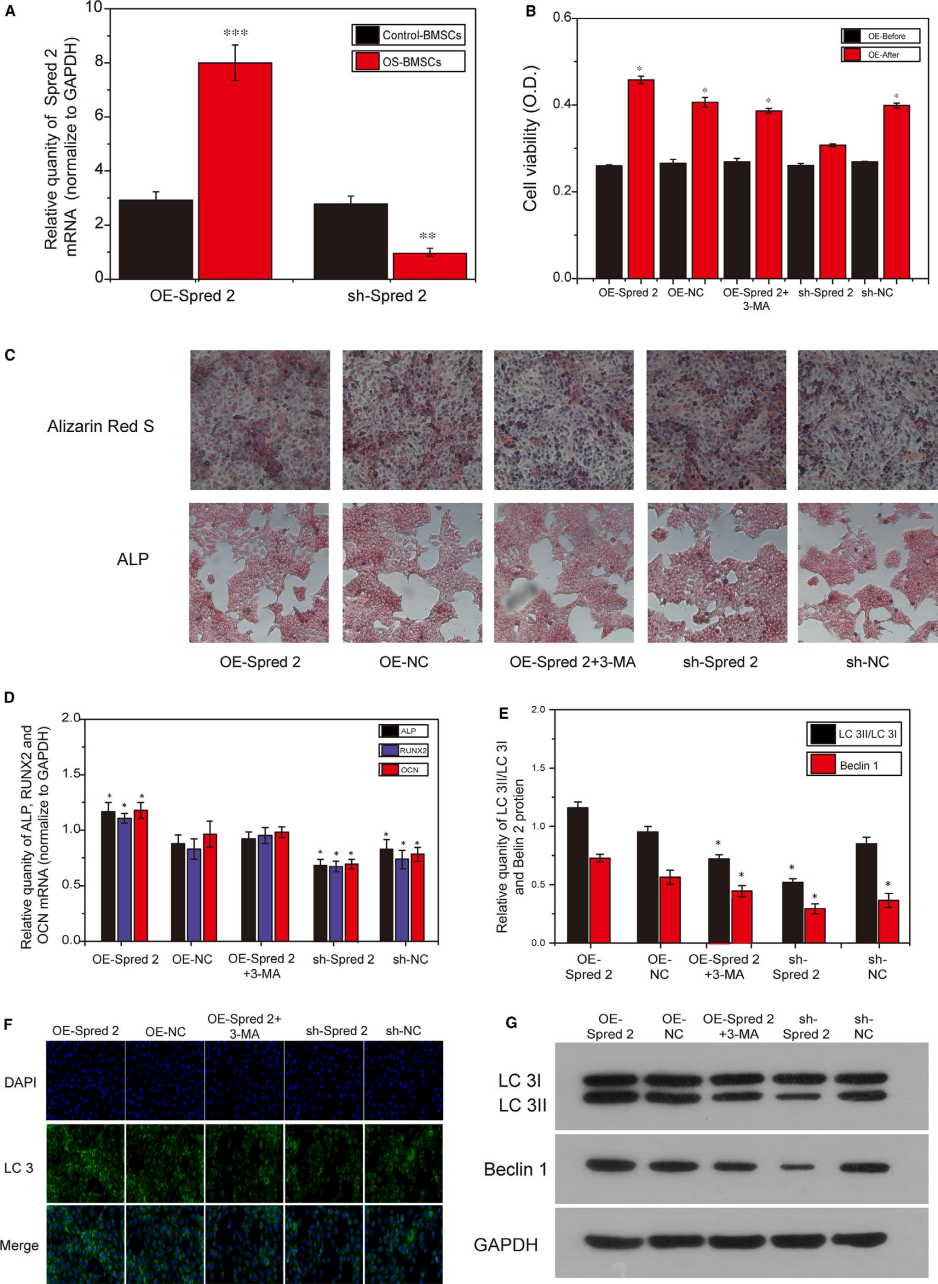
Spred2 promoted the activity, autophagy and osteogenic differentiation of BMSCs derived from OS rats by activating autophagy. A, The verification of overexpression and inhibition of Spred2. B, The activity of BMSCs detected by CCK 8. C, Alizarin Red S and ALP detection of osteogenic differentiation after OS‐BMSCs overexpression and inhibition of Spred2. D, PCR results of OS‐BMSCs osteogenic differentiation‐related genes ALP, RUNX2 and OCN. E, Western blotting detection of autophagy‐related proteins LC 3I/LC 3II. F, Statistical analysis result of LC 3I/LC 3II. G, Detection of LC 3 by immunofluorescence method. *, *P* < .05; **, *P* < .01; ***, *P* < .001

In order to explore the effect of Spred2 on BMSCs in the OS group, Spred2 was successfully overexpressed and inhibited (Figure 5A). The results of CCK8 showed that the cell activity of OE‐Spred2 was significantly higher than that of other groups, and using 3‐MA (autophagy inhibitor) resulted in a decrease in the BMSCs activity of OE‐Spred2 + 3‐MA (Figure 5B). Alizarin Red S and ALP stained calcium mineralization after osteogenic differentiation. The results showed that BMSCs in the OE‐Spred2 group had the highest osteogenic differentiation ability and high osteogenic differentiation‐related gene (ALP, RUNX2 and OCN) expression (Figure 6C,D), sh‐Spred2 group showed low calcium mineralization ability and decreased expression of genes related to osteogenic differentiation. Western blotting and immunofluorescence staining analysis results showed that LC 3I/LC 3II in the OE‐Spred2 group were significantly increased. On the contrary, after Spred2 was inhibited, the expression levels of LC 3I/LC 3II were significantly lower than those in the OE‐Spred2 (*P* < .05).

### 3.7 Spred2 inhibited cell cycle arrest and cell senescence of BMSCs derived from OS rats by activating autophagy

**FIGURE 7:**
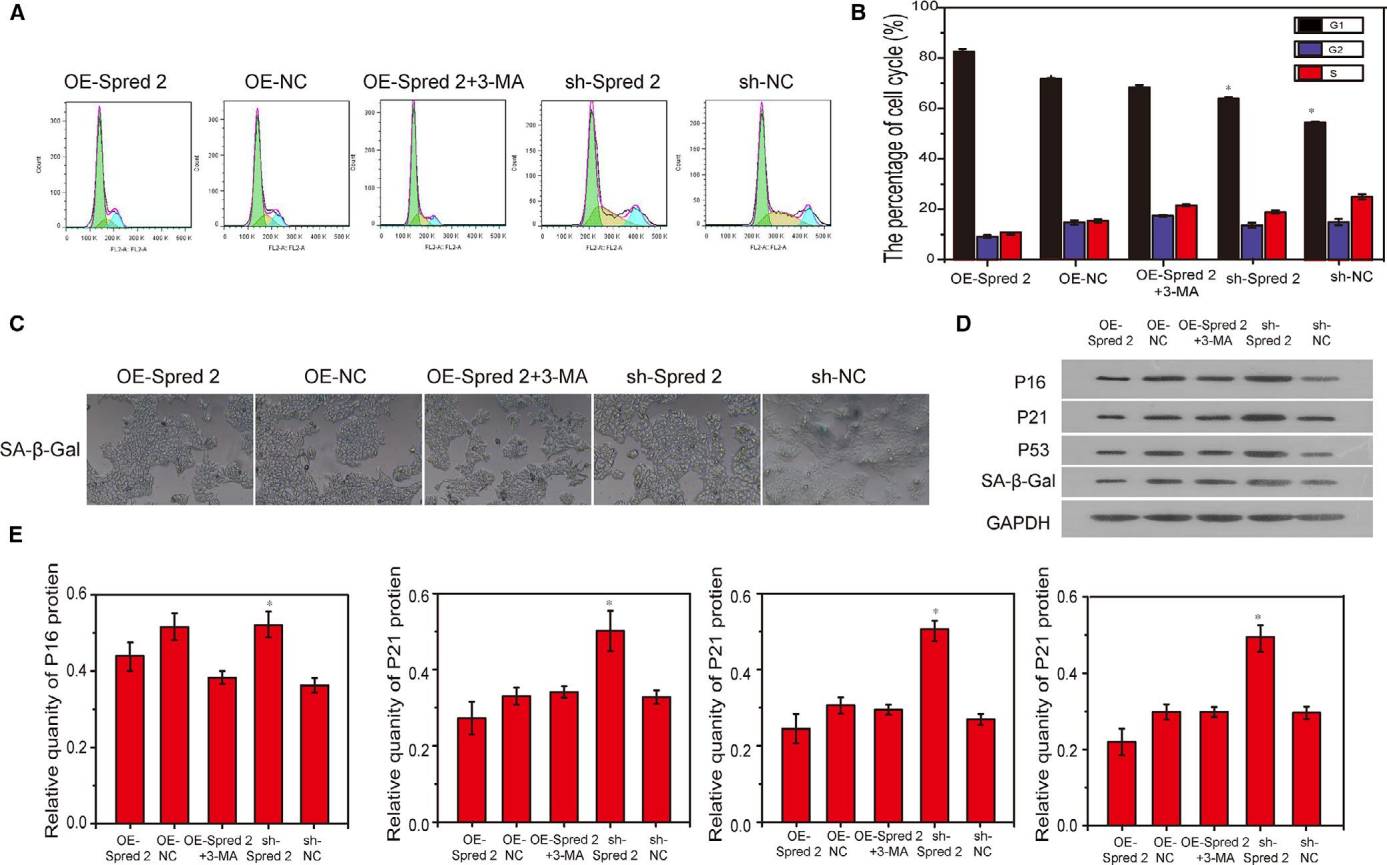
Spred2 inhibited cell cycle arrest and cell senescence of BMSCs derived from OS rats by activating autophagy. A, Flow cytometry detected the cell cycle of BMSCs in different groups. B, Statistical analysis of cell cycle. C, After overexpression and inhibition of Spred2, the senescence of different groups of cells were tested by SA‐β‐Gal staining. D, Western blotting detection of P16, P21 and P53 proteins in different groups. F, Statistical analysis of Western blotting results. *, *P* < .05; **, *P* < .01; ***, *P* < .001

In order to explore the effects of Spred2 on the cell cycle and senescence of BMSCs derived from OS rats, after overexpression and inhibition of Spred2, the results showed that the cell G1 phase and SA‐β‐Gal levels in OE‐Spred2 group were significantly higher than those of other groups. The G1 phase of BMSCs decreased, and the S phase increased in OE‐Spred2 + 3‐MA (Figure 7A,B). The S phase of the sh‐Spred2 group was as high as 30%. SA‐β‐Gal staining was used to detect the cell senescence, and the result was consistent with the flow cytometry results (Figure 7C). Subsequently, we detected the expression levels of P16, P21 and P53 proteins and their expression in the OE‐Spred2 group were the lowest and those in the sh‐Cav1.3 group were the highest (Figure 7D,E), indicating that spred2 activated cells autophagy by inhibiting cell cycle arrest and cell senescence of OS‐derived BMSCs.

## 4 DISCUSSION

OS is a decrease in bone mass caused by an imbalance between bone formation and bone resorption in the body. Bone formation is mainly regulated by osteoblasts. The proliferation of osteoblasts and the decline of bone formation ability greatly promote the occurrence of osteoporosis.^14^ The osteoblast cell line is derived from differentiation, and many studies have found that the gene expression in osteoporosis patients is different from that in normal control groups. Therefore, in this experiment, the whole bone marrow culture method was used to extract and purify rat BMSCs. The purpose was to study the effect of the relationship between Cav 1.3 and Spred 2 on the biological functions of OS rat BMSCs, and explore the specific mechanism of this effect.

At present, the identification of BMSCs is mainly based on the positive or negative expression of their specific surface markers, and the ability to target differentiation as a reference index.^15^ Therefore, we performed the identification of surface markers, osteogenic differentiation induction and the expression of osteogenic related genes of BMSCs. Our results demonstrated that the OS not only resulted reduced bone density and body weight, but also had a significantly higher calcium nodule positive than the control group. This indicated that the BMSCs of OS rats were successfully extracted in this study, and the reduced osteogenic differentiation ability of these BMSCs.

Cav 1.3 calcium channel protein is one of the important ion channel protein subunits to maintain the balance of intracellular Ca 2^+^ concentration. Its main role is to control intracellular Ca 2^+^ influx and depolarization of cell membranes.^16^ Several reports have shown that Cav1.3 calcium channels are associated with the maintenance of heart rhythm and age‐related hearing loss.^17^ And studies have found that Ca v 1.3 plays a role in cell differentiation, indicating that this protein may play a role in bone formation. But the mechanism is unclear. Our findings further prove the effect of Cav1.3 on the osteogenic differentiation of BMSCs (Alizarin Red S, ALP and qPCR results), and Cav 1.3 may affect the osteogenic differentiation of BMSCs through the interaction with Spred 2.

There is increasing evidence that cell senescence is related to multiple pathways, including cell division, shortening of telomeres, protein aggregation, DNA damage and cellular stress response caused by increased reactive oxygen species.^18^ These stress responses will further activate downstream p53/ p21 and other pathways, and produce a cascade reaction to trigger cell senescence.^19^ Cell senescence will cause the body's function to decline, and with the body's aging, the cell subgroups of various lineages in the bone microenvironment will age, which is mainly manifested by the decline of bone density and the reduction of the differentiation ability of BMSCs. In this study, the up‐regulated Cav1.3 was not only manifested as decreased osteogenic differentiation capacity of BMSCs, but also manifested as increased cellular senescence and the expression levels of P16, P21 and P53 proteins. This phenomenon was more obvious in the OS group.

Many studies have shown that Spred 2 is related to multiple pathways, and the interaction between Spred 2 and p85 affects the Ras/ERK pathway.^20^ The direct association of Spred 2 with DYRK1A modified the substrate/kinase interaction.^19^ Spred 2 exerts a negative regulatory effect on the differentiation of lens fibre cells, and it can induce caspase‐dependent and autophagy‐dependent cell death.^21^ This study found that overexpression of Cav 1.3 inhibited the autophagy ability of BMSCs in the OS group, manifesting as reduced expression of LC 3II/LC 3I and Beclin 1 proteins (autophagy‐related proteins). Furthermore, the BMSCs in the OS group not only decreased their autophagy ability, but also showed increased cell cycle arrest and cell senescence. Under normal physiological conditions, autophagy plays a key role in cell growth, development and homeostasis. In addition, autophagy is also related to cell proliferation, survival and senescence.^21^, ^22^, ^23^ At the same time, overexpression of Cav 1.3 and Spred 2 or inhibition of Cav 1.3 increased the level of cell autophagy. These results all indicated that the overexpressed Cav 1.3 may regulate autophagy by interacting with Spred 2, thereby inhibiting the proliferation, differentiation and senescence of BMSCs in the OS group.

To our knowledge, this is the first report showing that the interaction of Cav1.3 and Spred 2 regulates autophagy and participates in the biological functions of BMSC. In order to further confirm whether there is an interaction between Cav1.3 and Spred 2, GST‐pull down and CHIP we used to explore the interaction between Cav1.3 and Spred 2. The results show that there is a strong interaction between Cav1.3 and Spred 2, and this regulation is negatively correlated. Previous studies have not reported the relationship between Cav1.3 and Spred 2. This study is the first to report and clarify the relationship between the Cav1.3 and Spred 2. We further overexpressed Spred 2 to observe the improvement of the cell biological function of BMSCs in OS rats. Consistent with our expected results, after overexpression of Spred 2, the BMSCs viability and osteogenic differentiation ability of OS rats increased, while cell cycle arrest and cell senescence decreased, which means that Spred 2 promotes the proliferation and osteogenic differentiation of BMSCs in the OS group by activating autophagy.

This research has broad prospects and provides molecular biology guidance for basic research and clinical application of osteoporosis. This study also has limitations and no further animal experiments. In our next work, we will do more research on the animal level.

## 5 CONCLUSIONS

We systematically studied the influence of the relationship between Cav 1.3 and Spred 2 on the cell function of BMSCs. The increased Cav 1.3 in the OS group impaired the proliferation, differentiation and autophagy ability of BMSCs. Spred 2 can promote the proliferation, differentiation and autophagy of BMSCs, thereby slowing down the senescence of BMSCs. In short, Cav 1.3 negatively regulates Spred 2 mediated autophagy and cell senescence, and ultimately damages the activity and osteogenic differentiation of BMSCs in OS rats.

## CONFLICT OF INTEREST

All authors declare no conflict of interest.

## AUTHOR CONTRIBUTION

**Ping Fan:** Conceptualization (equal); Data curation (equal); Formal analysis (equal); Investigation (equal); Methodology (equal); Resources (equal); Software (equal); Supervision (equal); Validation (equal); Visualization (equal); Writing‐original draft (equal); Writing‐review & editing (equal). **Dan Pu:** Data curation (equal); Formal analysis (equal); Investigation (equal); Methodology (equal); Resources (equal); Software (equal); Supervision (equal); Validation (equal); Visualization (equal). **Xiaohong Lv:** Data curation (equal); Formal analysis (equal); Investigation (equal); Methodology (equal); Resources (equal); Software (equal); Supervision (equal); Validation (equal); Visualization (equal). **Nan Hu:** Formal analysis (equal); Investigation (equal); Methodology (equal); Resources (equal); Software (equal); Supervision (equal); Validation (equal); Visualization (equal). **Xiuyuan Feng:** Data curation (equal); Formal analysis (equal); Investigation (equal); Methodology (equal); Resources (equal); Software (equal); Supervision (equal). **Zhiming Hao:** Formal analysis (equal); Investigation (equal); Methodology (equal); Resources (equal); Software (equal); Supervision (equal). **Yining Sun:** Investigation (equal); Methodology (equal); Resources (equal); Software (equal). **Lan He:** Formal analysis (equal); Funding acquisition (equal); Investigation (equal); Project administration (equal); Resources (equal); Software (equal); Validation (equal); Visualization (equal).

## ETHICS APPROVAL AND CONSENT TO PARTICIPATE

The ethic approval was obtained from the Ethic Committee of the First Affiliated Hospital of Xi'an Jiaotong University School of Medicine.

## CONSENT TO PUBLISH

All of the authors have Consented to publish this research.

## Data Availability

The data are free access to available upon request.
